# Health-Related Quality of Life and Work Productivity of Adults With ADHD: A U.K. Web-Based Cross-Sectional Survey

**DOI:** 10.1177/1087054718799367

**Published:** 2018-09-14

**Authors:** Alain Joseph, Charlotte E. Kosmas, Chloe Patel, Helen Doll, Philip Asherson

**Affiliations:** 1 Shire, Zug, Switzerland; 2 ICON, Oxfordshire, UK; 3 King’s College London, Greater London, UK

**Keywords:** ADHD, adult, health-related quality of life, EQ-5D, WPAI:GH

## Abstract

**Objective:** The objective of this study was to assess health-related quality of life (HRQoL) in adult ADHD. **Method:** U.K. residents aged 18 to 55 years with ADHD and no major mental health comorbidities completed an online survey of disorder history, the EuroQoL 5-Dimensions 5-Level (EQ-5D-5L), and the Work Productivity and Activity Impairment Questionnaire: General Health (WPAI:GH). ADHD Rating Scale-IV (ADHD-RS-IV) score was assessed by telephone. **Results:** In total, 233 participants completed the study (mean age 32.6 years; 65.2% women). Mean (*SD*) ADHD-RS-IV total score, EQ-5D utility, and visual analog scale (VAS) scores were 43.5 (7.88), 0.74 (.21), and 69.8 (17.76), respectively. Mean (*SD*) WPAI:GH scores indicated that health problems caused 45.7% (29.9) overall work impairment and 45.8% (28.9) impairment in regular daily activities. Greater work and activity impairment were both significantly independently associated with lower utility after adjusting for age, gender, and somatic comorbidities. **Conclusion**: Adult ADHD impairs HRQoL, work productivity, and regular daily activities.

## Introduction

Recognized as one of the most common psychiatric disorders among children and adolescents, ADHD is also known to persist into adulthood in approximately two thirds of cases (Cheung et al., 2016; Faraone, Biederman, & Mick, 2006; Kooij et al., 2010). Indeed, ADHD is estimated to affect around 3% to 4% of adults worldwide (de Graaf et al., 2008; Fayyad et al., 2007; Kessler et al., 2006). While the disorder is characterized by the core symptoms of hyperactivity, impulsivity, and inattention, ADHD diagnosis also requires significant psychological, social, and/or educational or occupational impairment (American Psychiatric Association, 2013; National Institute for Health and Care Excellence [NICE], 2008a). The impairments associated with ADHD evolve as an individual matures and as in adulthood the need for personal organization increases, the disorder often presents as internal restlessness, impatience, distractibility, disorganization, forgetfulness, and poor timekeeping (Asherson, Manor, & Huss, 2014). Emotional dysregulation (irritability, mood swings, and volatile temper outbursts) is also common (Asherson et al., 2014). In addition, adult ADHD is associated with a wide range of psychosocial impairments (Brod, Pohlman, Lasser, & Hodgkins, 2012; Pitts, Mangle, & Asherson, 2015; Ramos-Quiroga, Montoya, Kutzelnigg, Deberdt, & Sobanski, 2013) including lower educational attainment and poorer workplace performance (de Graaf et al., 2008; Ebejer et al., 2012; Kessler et al., 2005), and difficulties establishing and sustaining close personal relationships (Biederman, Faraone et al., 2006; Brod, Schmitt, Goodwin, Hodgkins, & Niebler, 2012; Das, Cherbuin, Butterworth, Anstey, & Easteal, 2012; Pitts et al., 2015). In the family environment, some adults with ADHD lack appropriate parenting techniques and report more family conflicts (Biederman, Faraone, & Monuteaux, 2002; Brod, Pohlman, et al., 2012; Chronis-Tuscano et al., 2008).

Studies conducted in Canada, Europe, Israel, Taiwan, and the United States indicate that ADHD adversely affects health-related quality of life (HRQoL; Chao et al., 2008; Gjervan, Torgersen, & Hjemdal, 2016; Grenwald-Mayes, 2002; Karlsdotter et al., 2016; Lensing, Zeiner, Sandvik, & Opjordsmoen, 2015; Rimmerman, Yurkevich, Birger, Azaiza, & Elyashar, 2007; van Hout et al., 2012) in multiple domains. These include life productivity, psychological health, relationships, life outlook (Adler et al., 2013; Brod, Johnston, Able, & Swindle, 2006), work productivity (Brod, Schmitt, et al., 2012), psychosocial well-being (Matza et al., 2004), life enjoyment and satisfaction (Mick, Faraone, Spencer, Zhang, & Biederman, 2008). Few studies, however, have assessed the impact of ADHD on health utility in adults—a disease-independent measure of HRQoL commonly used to inform guideline development and decision-making. Those studies that have assessed health utility recruited populations with a high rate of psychiatric comorbidities, making it difficult to identify the independent impact of ADHD (Karlsdotter et al., 2016; Lensing et al., 2015; van Hout et al., 2012).

Here we present the results of a web-based survey of adults resident in the United Kingdom who report a diagnosis of ADHD and no major comorbid mental health disorders. The study was designed to evaluate the impact of ADHD on HRQoL using the EuroQoL 5-Dimensions 5-Level (EQ-5D-5L), and on work and regular daily activities using the Work Productivity and Activity Impairment Questionnaire: General Health (WPAI:GH) assessment.

## Method

### Study Design Overview

Adults reporting a formal diagnosis of ADHD whose responses to a self-completed online ADHD assessment were aligned with *Diagnostic and Statistical Manual of Mental Disorders* (5th ed.; *DSM-5*; American Psychiatric Association, 2013) criteria for adult ADHD participated in an online survey and subsequent telephone interview. Information requested from participants in the online survey included sociodemographic characteristics, ADHD disorder history, chronic medical (somatic) comorbidities, EQ-5D-5L responses, and WPAI:GH assessment. ADHD severity was assessed using the ADHD Rating Scale-IV (ADHD-RS-IV) with adult prompts administered by a trained interviewer via telephone.

The study protocol and survey materials were approved by an independent review board before study initiation to cover data collection in the United Kingdom (Salus institutional review board, protocol number 0238-0256, approved February 19, 2015). Informed consent was collected online and all data were anonymized before analysis.

Recruitment of a sample of 300 adults was planned to allow for the identification of significant differences, at the 5% level (α = .05) and with 80% power, between two groups of participants of equal size equivalent to a moderate effect size of ≈0.3 *SDs* (Cohen, 1988) and, in the whole sample, to provide a confidence interval (CI) of maximum width ±5% around a percentage.

### Participants and Screening

Eligible participants were aged 18 to 55 years, reported a formal diagnosis of ADHD, were resident in the United Kingdom, and were able to give informed consent. A specialist patient research and fieldwork agency (Opinion Health, London, UK) used these criteria to identify and contact (via email) individuals from their patient community who had previously provided consent to be contacted should they be potentially eligible for inclusion in a study. These individuals had been recruited through patient associations or through a range of traditional recruitment activities. Study participants received a £30 voucher or check as reimbursement for their time.

Screening consisted of an online form to determine study eligibility followed by a self-completed online ADHD assessment. Patients were excluded if they self-reported one or more of the following comorbid major mental health disorders, presented as a checklist on the screening form: Asperger’s syndrome, autism, schizophrenia, psychosis, severe depression/mania, drug addiction, severe anxiety disorder, and obsessive compulsive disorder. Identity checks were conducted during the screening process, via Internet protocol (IP) address, to ensure that each participant was resident in the United Kingdom and completed screening only once. The self-completed online ADHD assessment was used for screening purposes only and included questions about participants’ ADHD symptoms (aligned with the 18 *Diagnostic and Statistical Manual of Mental Disorders* (4th ed.; *DSM-IV*; American Psychiatric Association, 1994)*/DSM-5* adult ADHD items) along with questions about how long these challenges had been present, and their impact on multiple areas of daily life. Patients whose responses were consistent with the *DSM-5* criteria for adult ADHD (American Psychiatric Association, 2013), including onset before starting secondary school, were directed to the online survey.

### The Survey

The survey comprised four main components: (a) the participant description; (b) the EQ-5D-5L; (c) the WPAI:GH; and (d) the ADHD-RS-IV (telephone interview).

The participant description comprised questions regarding sociodemographic characteristics (age, gender, ethnicity, highest level of education, employment status) and ADHD disorder history (age of first ADHD diagnosis, type of medical professional service making first diagnosis, age of a more recent ADHD diagnosis [if applicable], age of perceived ADHD symptom onset, current use of ADHD medication [yes/no], current use of nonpharmacological ADHD treatment [checklist]). Participants also filled in a checklist to report on the presence of common chronic medical (somatic) comorbidities known to affect HRQoL: asthma, angina, cancer, chronic heart disease, chronic obstructive pulmonary disease, chronic renal disease, diabetes, rheumatoid arthritis, and other.

The EQ-5D is a generic preference-based measure of health status that has been validated as a practical tool to assess HRQoL in the general population and many patient groups (EuroQol Group, 1990; Herdman et al., 2011; Kind, Dolan, Gudex, & Williams, 1998; Peters, Crocker, Jenkinson, Doll, & Fitzpatrick, 2014). The EQ-5D classifies health state across five domains: mobility, self-care, usual activities (work, study, housework, family, and leisure), pain/discomfort, and anxiety/depression. For each domain, the EQ-5D-5L instrument (EuroQol Group, 2009) includes five possible levels of severity resulting in 3,125 possible combinations or health states (Herdman et al., 2011). Each health state is allocated a health “utility” value on a scale anchored at 1 (*best possible health*) and 0 (*dead*), which is weighted using value sets that represent the preferences of the general population (Devlin & Krabbe, 2013). In the present study, EQ-5D-5L responses were converted to utilities using the validated EuroQol mapping (crosswalk) function (van Hout et al., 2012) and established U.K. preference values (Dolan, Gudex, Kind, & Williams, 1995). The EQ-5D-5L also incorporates a visual analog scale (VAS) for respondents to rate their health from 100 (*best health you can imagine*) to 0 (*worst health you can imagine*) (EuroQol Group, 2009). Thus, a low EQ-5D utility or VAS score indicates poor preference for a health state.

The WPAI:GH is a validated instrument that measures the effect of health problems, defined as any physical or emotional problem or symptom, on work productivity and regular daily activities within the previous 7 days (Reilly, Zbrozek, & Dukes, 1993). The four outcome measures quantify the effect of health problems on proportion of work time missed, percentage impairment while working, percentage overall work impairment (a combination of the “work time missed” and “impairment while working” measures), and percentage impairment on ability to do regular daily activities. Individuals reporting that they are not “currently employed (working for pay)” are assessed only on the activity impairment measure (Reilly Associates, 2004).

The severity of participants’ ADHD symptoms was assessed by trained interviewers via telephone using the ADHD-RS-IV with adult prompts. The ADHD-RS-IV consists of the 18 *DSM-IV/5* items: nine for inattentiveness symptoms and nine for hyperactive/impulsive symptoms. The severity of each symptom is rated on a 4-point scale from 0 (*none*) to 3 (*severe*), and these values are summed to give the total score.

### Data Analysis

In descriptive analyses, responses to individual questions were summarized using means with *SD*, and response frequencies as *n* (%). The uncertainty in the sample estimates is captured by 95% CIs. In inferential analyses to examine factors associated with ADHD-RS-IV total scores, EQ-5D utilities, EQ-5D VAS scores, and WPAI:GH outcomes, significance was assessed using *t* tests or analyses of variance, as appropriate. Pearson correlation coefficients were used to assess the relationships between continuous variables. Linear regression was used to adjust for the possible confounding variables of age, gender, and presence of chronic medical comorbidities. These possibly confounding variables were entered first in each model, with subsequent variables added in a stepwise fashion. Significance was taken throughout at the 5% level (α = .05); however, nominal significance levels at *p* < .01 rather than *p* < .05 are considered most reliable owing to the number of tests performed and the descriptive aims of this research. Analyses were conducted in SPSS v19.0 and Stata v14.0, data were cleaned before analysis, and no data imputation was performed.

## Results

### Study Population and Sociodemographic Characteristics

**Figure 1.**
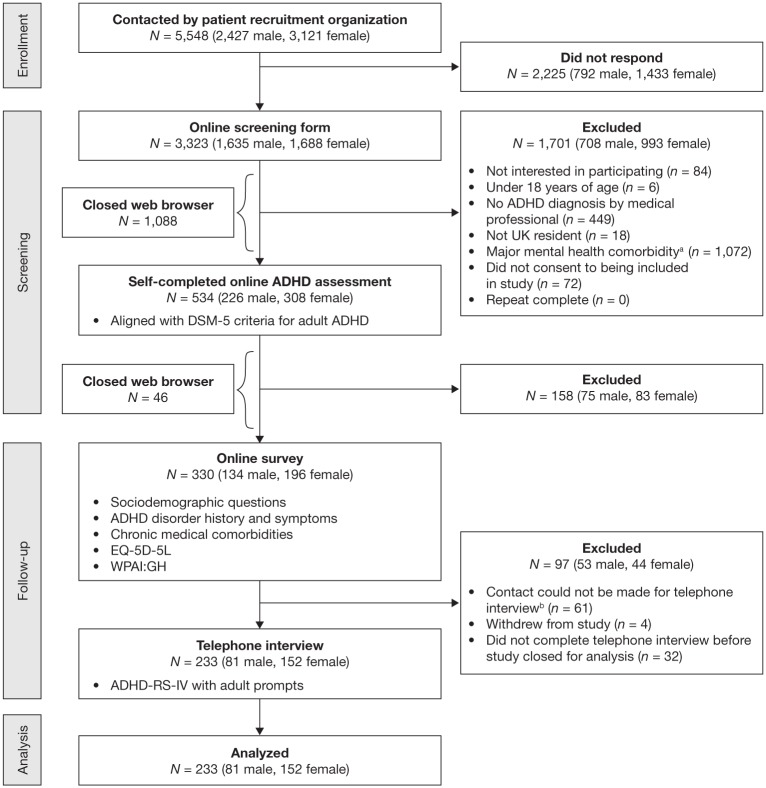
Participant flow. *Note. DSM-5* = *Diagnostic and Statistical Manual of Mental Disorders, Fifth Edition*; EQ-5D-5L = 5-level 5-dimensions EuroQol questionnaire; WPAI:GH = Work Productivity and Activity Impairment Questionnaire: General Health; ADHD-RS-IV = ADHD Rating Scale-IV. ^a^Presented as a checklist: Asperger’s syndrome, autism, schizophrenia, psychosis, severe depression/mania, drug addiction, severe anxiety disorder, obsessive compulsive disorder, none of the above. ^b^For example, incorrect telephone number provided, participant did not respond when called on several occasions.

The study took place from June 3, 2015, to October 30, 2015; a diagram of participant flow through enrollment, screening, follow-up, and analysis is shown in Figure 1. The initial screening form was accessed by 3,323 individuals. Of these, 534 were included and proceeded to the next stage, with 1,701 not meeting inclusion criteria and 1,088 closing their web browser prematurely. The most common reasons for exclusion were reporting a major mental health comorbidity (*n* = 1,072) and not having an ADHD diagnosis from a medical professional (*n* = 449). Of the 534 individuals who met the initial inclusion criteria and accessed the online ADHD assessment tool, 158 were excluded due to responses that were not consistent with the *DSM-5* criteria for adult ADHD and 46 closed their web browser prematurely. The online survey was completed by 330 individuals, of whom 97 were subsequently excluded for not completing the telephone interview (*n* = 93) or withdrawing from the study (*n* = 4).

The final sample consisted of 233 participants who completed the online survey and telephone interview. The sociodemographic characteristics of the 233 participants are summarized in Table 1. The mean age of participants was 32.6 years (*SD* = 9.5), the majority were women (*n* = 152) and most were of White British ethnicity (*n* = 180). Approximately one third of participants had completed a university course (*n* = 78), and one third were in full-time employment (*n* = 80). All surveys and tools were completed in full by the 233 participants.

**Table 1.** Sociodemographic Characteristics (*N* = 233).

Characteristic	
Gender, *n* (%)	
Male	81 (34.8)
Female	152 (65.2)
Mean (*SD*) age, years	32.6 (9.5)
Ethnicity, *n* (%)	
White British	180 (77.3)
Any other White background	22 (9.4)
White and Black Caribbean	4 (1.7)
White and Asian	4 (1.7)
Indian	7 (3.0)
African	5 (2.1)
Other/prefer not to answer	11 (4.7)
Level of education, *n* (%)	
No formal qualifications	15 (6.4)
Left school aged 16 years with qualifications	30 (12.9)
Left school aged 18 years with qualifications	25 (10.7)
Technical/vocational qualifications from a college or job	51 (21.9)
Completed university	78 (33.5)
Other/prefer not to answer	34 (14.6)
Employment status, *n* (%)	
Working full-time	80 (34.3)
Working part-time	33 (14.2)
Self-employed	26 (11.2)
Employed, currently off on long-term sick leave	3 (1.3)
Running household (not employed)	18 (7.7)
Early retirement due to ADHD	3 (1.3)
Seeking work, unemployed	15 (6.4)
Disabled	2 (0.9)
Full-time student	24 (10.3)
Temporarily prevented from working by sickness/injury	7 (3.0)
Permanently unable to work because of long-term sickness/disability	9 (3.9)
Other/prefer not to answer	13 (5.6)

### Clinical Characteristics and Disorder History

Clinical characteristics and disorder history are summarized in Table 2. The mean age of initial ADHD diagnosis was 22.98 years (*SD* = 13.1). Overall, 51.5% of participants were currently taking medications for ADHD. Some participants were also receiving nonpharmacological ADHD treatment (often in addition to medication), most commonly behavioral therapy (*n* = 19) and individual counseling (*n* = 18). Approximately 40% of participants reported at least one chronic medical comorbidity, the most common of which was asthma (*n* = 48). In the survey, most participants (87.6%) reported experiencing their first symptoms of ADHD before the age of 13 years. At screening, however, all 233 participants had reported that symptoms had been present since primary school (typically completed before 12 years of age in the UK; Government Digital Service, 2017a, 2017b).

**Table 2.** Clinical Characteristics and Disorder History (*N* = 233).

Characteristic	
Age at perceived symptom onset, years, *n* (%)	
0-7	148 (63.5)
8-12	56 (24.0)
13-17^a^	21 (9.0)
18 and over^a^	8 (3.4)
Age at first ADHD diagnosis, years, mean (*SD*)	22.98 (13.1)
Diagnosis by medical professional service type, *n* (%)	
Pediatric service	14 (6.0)
Child psychiatrist service	59 (25.3)
Adult psychiatrist service	101 (43.3)
Primary care clinic	25 (10.7)
Psychology service	17 (7.3)
Other	17 (7.3)
Age at second ADHD diagnosis, years, mean (*SD*) (*n* = 53)^b^	27.17 (9.4)
Currently receiving medication for ADHD, *n* (%)	
No	113 (48.5)
Yes	120 (51.5)
Currently receiving nonpharmacological ADHD treatment (*n* = 44), *n* (%)^c^	
Behavioral therapy (including CBT [*n* = 19] and neurofeedback [*n* = 1])	20 (8.6)
Individual counseling (*n* = 18) or coaching (*n* = 7)	25 (10.7)
Exercise program	4 (1.7)
Meditation/mindfulness	10 (4.3)
Dietary/nutritional changes	6 (2.6)
Other	11 (4.7)
Number of chronic medical comorbidities, *n* (%)	
0	139 (59.7)
1	79 (33.9)
2	12 (5.2)
3	3 (1.3)
Chronic medical comorbidities, *n* (%)^c^	
Asthma	48 (20.6)
Angina	3 (1.3)
Chronic heart disease	1 (0.4)
Chronic renal disease	0
COPD	0
Cancer	0
Diabetes	4 (1.7)
Rheumatoid arthritis	7 (3.0)
Other	49 (21.0)
No other health problems	134 (57.5)
Prefer not to answer	5 (2.1)

*Note.* Percentages may not total 100% owing to rounding. CBT = cognitive behavioral therapy; COPD = chronic obstructive pulmonary disease.

a All participants stated during screening that symptoms of adult ADHD had started before secondary school but some subsequently reported an older age in response to the survey question: “At what age do you think your ADHD symptoms first started?”

b Participants who responded positively to the survey question: “Have you had another more recent diagnosis of ADHD since your first diagnosis?”

c Not mutually exclusive.

### ADHD Symptom Severity

The mean ADHD-RS-IV total score was 43.46 (*SD* = 7.88) and was significantly higher in women (44.22 [*SD* = 7.77]) than in men (41.77 [*SD* = 7.91]) (*p* = .023). When ADHD-RS-IV total scores were compared between groups of participants classified by sociodemographic or disease characteristics, greater symptom severity (higher ADHD-RS-IV total score) was also significantly associated with not working (*p* = .001), not completing school to age 18 years or not completing university (*p* = .005), currently receiving nonpharmacological ADHD treatment (*p* = .037), and receiving a first ADHD diagnosis from a psychiatry, psychology, or other service (vs. a primary care clinic or pediatric service, *p* < .001) (Table 3). No significant associations were found between ADHD-RS-IV total scores and age category, number of medical comorbidities, currently taking medication for ADHD (vs. not), or age at first or second ADHD diagnosis (data not shown).

**Table 3.** Sociodemographic and Clinical Factors Significantly Associated With ADHD-RS-IV Total Scores.

Binary variables	*M*	*SD*	*M* (*SE*) difference	95% CI	*p* value
Gender					
Male, *n* = 81	41.77	7.91	2.45 (1.08)	[0.33, 4.57]	.023
Female, *n* = 152	44.22	7.77			
Employment status					
Working full/part-time, self-employed, *n* = 139	41.96	8.16	3.47 (1.01)	[1.44, 5.50]	.001
Not working, *n* = 94	45.44	7.01			
Level of education					
Completed school aged 18 years/university, *n* = 103	41.76	8.38	2.88 (1.03)	[0.86, 4.90]	.005
Other, *n* = 130	44.64	7.25			
Nonpharmacological ADHD treatment currently received					
No, *n* = 189	42.85	8.13	2.74 (1.31)	[0.16, 5.33]	.037
Yes, *n* = 44	45.59	6.33			
Diagnosis by medical professional service type					
Pediatric service/primary care clinic, *n* = 39	38.82	9.72	5.46 (1.34)	[2.82, 8.10]	< .001
Psychiatry/psychology service/other, *n* = 194				44.28	7.15
Categorical variables	Mean	*SD*			*p* value
Diagnosis by medical professional service type					
Pediatric service, *n* = 14	38.07	8.61	vs. Adult psychiatry service *p* = .038		.004
Child psychiatry service, *n* = 59	44.34	7.57			
Adult psychiatry service, *n* = 101	44.57	6.98			
Primary care clinic, *n* = 25	39.24	10.44	vs. Adult psychiatry service *p* = .025		
Psychology service, *n* = 17	42.24	8.26			
Other, *n* = 17	44.35	5.66			
Age of perceived symptom onset					
0 to 7 years, *n* = 148	44.06	7.09			< .001
8 to 12 years, *n* = 56	43.64	7.47			
13 to 17 years, *n* = 21	42.10	9.59			
18 years and over, *n* = 8	31.88	11.74			

*Note.* Owing to the number of tests performed and the descriptive aims of this research, nominal significance levels at *p* < .01 rather than *p* < .05 are considered most reliable. No significant associations were found between ADHD-RS-IV total scores and age category, number of medical comorbidities, currently taking medication for ADHD (vs. not), or age at first or second ADHD diagnosis. ADHD-RS-IV = ADHD Rating Scale-IV; CI = confidence interval.

### Health-Related Quality of Life Assessment: EQ-5D

Overall, mean EQ-5D utility and VAS scores were 0.74 (*SD* = 0.21) and 69.81 (*SD* = 17.76), respectively. These scores are qualitatively lower than the U.K. population norms for adults aged 18 years and over (0.86 [*SD* = 0.23] and 82.48 [*SD* = 16.96], respectively) and similar to those reported by adults aged 75 years and over (Kind, Hardman, & Macran, 1999).

Lower utilities (poorer HRQoL) were significantly associated with greater ADHD symptom severity (higher ADHD-RS-IV total scores, *r* = −.225, *p* < .001). Lower utilities were also significantly associated with lower work productivity due to health problems (higher work time missed due to health problems [*r* = −.183, *p* = .031], greater impairment while working due to health problems [*r* = −.431, *p* < .001], and greater overall work impairment due to health problems [*r* = −.424, *p* < .001]), greater activity impairment due to health problems (*r* = −.449, *p* < .001), and older ages at first and second ADHD diagnosis (*r* = −.13, *p* = .040 and *r* = −.27, *p* = .049, respectively). When utilities were compared between groups of participants classified by sociodemographic or disease characteristics, lower utilities (poorer HRQoL) were significantly associated with not working (*p* < .001); having chronic medical comorbidities (vs. not, *p* < .001); receiving a first ADHD diagnosis from a psychiatry, psychology, or other service (vs. a primary care clinic or pediatric service, *p* < .001); not completing school to age 18 years or not completing university (*p* = .033); and older age (*p* = .012).

Lower EQ-5D VAS scores were associated with greater WPAI:GH activity impairment due to health problems (*r* = −.328, *p* < .001), more work time missed due to health problems (*r* = −.179, *p* = .034), greater impairment while working due to health problems (*r* = −.237, *p* = .006), and greater overall work impairment due to health problems (*r* = −.250, *p* = .004). When EQ-5D VAS scores were compared between groups of participants classified by sociodemographic or disease characteristics, lower scores were significantly associated with not working (*p* < .001) and with having chronic medical comorbidities (vs. not, *p* < .001).

After controlling for age, gender, and the presence of chronic medical comorbidities, the following significant independent associations were observed: greater activity impairments due to health problems were associated with lower EQ-5D utilities (*p* = .006) and VAS scores (*p* < .001), and greater overall work impairments due to health problems were associated with lower EQ-5D utilities (*p* = .033). The regression models indicated that, after controlling for all other factors, utility decreased by .05 points for each additional decade of age (*p* = .001), men had lower utilities than women (by .089 points, *p* = .001), and participants who reported at least one chronic medical comorbidity had lower EQ5D VAS scores than those who did not (by 8.588 points, *p* < .001; Table 4).

**Table 4.** Regression Models/ANOVAs to Identify Factors Independently Associated With EQ-5D Utility and VAS Scores.

	EQ-5D utility^a^				EQ-5D VAS score			
	*B*	*SE*	*t*	*p* value	*B*	*SE*	*t*	*p* value
(Constant)	0.977	0.061	16.02	< .001	81.30	5.168	15.73	< .001
Age, years	−0.005	0.002	−3.53	.001	−0.177	0.114	−1.55	.122
Gender, female	0.089	0.026	3.35	.001	3.425	2.268	1.51	.132
Chronic medical comorbidity^b^	−0.052	0.027	−1.92	.057	−8.588	2.234	−3.84	< .001
% overall work impairment due to health problems	−0.001	0.001	−2.15	.033	−	−	−	−
% activity impairment due to health problems	−0.002	0.001	−2.82	.006	−0.173	0.038	−4.55	< .001

*Note.* Only significant associations after adjusting for age, gender, and the presence of chronic medical comorbidities are shown. ANOVAs = analyses of variance; EQ-5D-5L = 5-dimension 5-level EuroQol questionnaire; VAS = visual analog scale; *B* = regression coefficient; *t* = *t* statistic for assessing significance.

a EQ-5D-5L responses were converted to utilities using the validated EuroQol mapping function (van Hout et al., 2012) and UK preference values (Dolan, Gudex, Kind, & Williams, 1995).

b One or more of asthma, angina, cancer, chronic heart disease, chronic obstructive pulmonary disease, chronic renal disease, diabetes, rheumatoid arthritis or other (vs. none).

### Productivity Assessment: WPAI:GH

Mean impairment due to health problems of regular daily activities was 45.79% (*SD* = 28.86) in the overall sample of 233 individuals. The 135 participants who indicated that they were “currently employed (working for pay)” on the WPAI:GH instrument reported a mean proportion of work time missed due to health problems of 15.71% (*SD* = 26.48), a mean impairment while working due to health problems of 40.59% (*SD* = 27.01), and a mean overall work impairment due to health problems of 45.65% (*SD* = 29.86).

Significantly greater activity impairment due to health problems was associated with greater ADHD symptom severity (higher ADHD-RS-IV total score, *r* = .256, *p* < .001); not working (*p* = .011); not currently taking ADHD medication (*p* = .027); receiving a first ADHD diagnosis from a psychiatry, psychology, or other service (vs. a primary care clinic or pediatric service, *p* = .009); and older age at first ADHD diagnosis (*r* = .209, *p* = .001). When WPAI:GH outcomes were compared between groups of participants classified by sociodemographic or disease characteristics, women reported that health problems caused significantly more impairment while working (*p* = .025) and of regular daily activities (*p* = .032) than did men. Participants with at least one chronic medical comorbidity reported that health problems caused significantly more impairment while working (*p* = .032), overall work impairment (*p* = .024), and activity impairment (*p* = .003) than those reporting no medical comorbidities. Associations between WPAI:GH and EQ-5D outcomes have already been reported in the previous section.

Regression analyses adjusting for age, gender, and the presence of chronic medical comorbidities were conducted to identify factors significantly independently associated with WPAI:GH outcomes. For these analyses, EQ5D utility was categorized in quartiles owing to nonnormality of the data distribution (lowest quartile below .68, *n* = 62; second quartile .68 to .78, *n* = 57; third quartile .785 to .85, *n* = 57; fourth quartile above .85, *n* = 57). Each of the four WPAI:GH outcomes was significantly independently associated with lower EQ-5D utility quartile (*p* = .033 for work time missed due to health problems; *p* < .001 for the other outcomes). In addition, more work time missed due to health problems was significantly independently associated with younger age at second ADHD diagnosis (*p* = .010). The regression models indicated that, after controlling for all other factors, women reported that health problems caused more impairment while working, overall work impairment, and impairment of regular daily activities than did men (by 14.12 [*p* = .002], 11.88 [*p* = .020], and 8.748 [*p* = .018] percentage points, respectively; Table 5).

**Table 5.** Regression Models/ANOVAs to Identify Factors Independently Associated With WPAI:GH Scores: Work-Related Outcomes, *n* = 135 (A) and Activity-Related Outcomes, *N* = 233 (B).

A	% work time missed due to health problems				% impairment while working due to health problems				% overall work impairment due to health problems			
	*B*	*SE*	*t*	*p* value	*B*	*SE*	*t*	*p* value	*B*	*SE*	*t*	*p* value
(Constant)	25.949	23.54	1.10	.280	58.30	12.37	4.71	< .001	70.11	13.83	5.07	< .001
Age, years	1.255	0.796	1.58	.126	−0.416	0.27	−1.52	.132	−0.461	0.31	−1.50	.135
Gender, female	5.886	8.041	0.73	.470	14.12	4.52	3.12	.002	11.88	5.06	2.35	.020
Chronic medical comorbidity^a^	8.156	6.336	1.29	.209	3.628	4.58	0.79	.430	5.433	5.12	1.06	.291
Age at receiving second ADHD diagnosis	−1.755	0.635	−2.77	.010	−	−	−	−	−	−	−	−
EQ-5D utility quartile^b,c^	−7.861	3.498	−2.25	.033	−10.31	2.00	−5.16	< .001	−11.16	2.23	−5.00	< .001
B	% activity impairment due to health problems											
	*B*				*SE*				*t*		*p*-value	
(Constant)	49.03				9.52				5.15		< .001	
Age, years	0.118				0.186				0.63		.528	
Gender, female	8.748				3.655				2.39		.018	
Any chronic medical comorbidity^a^	5.054				3.647				1.39		.167	
EQ-5D utility quartile^b,c^	−9.545				1.595				−5.98		< .001	

*Note.* Only significant associations after adjusting for age, gender and the presence of chronic medical comorbidities are shown. ANOVAs = analyses of variance; WPAI:GH = Work Productivity and Activity Impairment Questionnaire: General Health; *B* = regression coefficient; *t* = *t* statistic for assessing significance; EQ-5D-5L = 5-dimension 5-level EuroQol questionnaire.

a One or more of asthma, angina, cancer, chronic heart disease, chronic obstructive pulmonary disease, chronic renal disease, diabetes, rheumatoid arthritis or other (vs. none).

b EQ-5D-5L responses were converted to utilities using the validated EuroQol mapping function (van Hout et al., 2012) and UK preference values Dolan, Gudex, Kind, and Williams (1995).

c For these analyses EQ-5D utility was categorized in quartiles because of nonnormality of the data distribution: lowest quartile below .68 (*n* = 62); second quartile .68 to .78 (*n* = 57); third quartile .785 to .85 (*n* = 57); fourth quartile above .85 (*n* = 57).

## Discussion

The present study aimed to clarify the impact of ADHD on HRQoL and impairments in work and regular daily activities in a sample of 233 U.K. residents aged 18 to 55 years with ADHD and no comorbid major mental health disorders. In the sample, more severe ADHD symptoms were significantly associated with poorer HRQoL (lower utilities) and greater health-related impairments in regular daily activities. Observed mean EQ-5D utility and VAS scores were lower than UK norms for the general population and similar to those reported by adults aged 75 years and over (Kind et al., 1999). Participants also reported that health problems led to considerable impairment in work and in regular daily activities.

HRQoL is a multidimensional construct of an individual’s perception of the impact of his or her health status on physical, psychological, and social functioning. Several studies around the world have investigated HRQoL in adults with ADHD, with impacts of ADHD on functioning and HRQoL in adults reported to be similar in several European and North American countries (Adler et al., 2013; Brod et al., 2006; Brod, Schmitt, et al., 2012; Chao et al., 2008; Gjervan et al., 2016; Grenwald-Mayes, 2002; Karlsdotter et al., 2016; Lensing et al., 2015; Matza et al., 2004; Mick et al., 2008; Rimmerman et al., 2007). Previous studies in which the impact of ADHD on health utility in adults was assessed, however, recruited populations with a high rate of psychiatric comorbidities, making it difficult to identify the independent impact of the disorder (Karlsdotter et al., 2016; Lensing et al., 2015; van Hout et al., 2012). Health utilities are important because they can be used to estimate quality-adjusted life-years, which are central to guideline development and decision-making for health care provision. The U.K. NICE (NICE) states that, for appraisals of health technologies (e.g., medications and devices), the EQ-5D is the preferred measure of HRQoL and utility in adults, and that other measures should be mapped to EQ-5D values (NICE, 2013).

The use of a single generic HRQoL instrument enables different health states, medical conditions, and interventions to be compared. In the present study, EQ-5D-5L responses were converted to utility values using the validated EuroQol 5L to three-level (3L) mapping function (van Hout et al., 2012) and established U.K. preference values (Dolan et al., 1995), as recommended by NICE (2013). This enables comparisons to be made between the present results and those of previous studies that used the original EQ5D[3L] questionnaire. Population norm EQ-5D data for a representative sample of 3,395 U.K. adults (Kind et al., 1999) has previously been developed using the same U.K. preference values and the original three-level questionnaire (Dolan et al., 1995). The present mean EQ-5D utility and VAS scores for U.K. adults with ADHD aged 18 to 55 years (0.74 and 69.81, respectively) are qualitatively lower than these U.K. population norms in adults aged 18 years and over (0.86 [*SD* = 0.23] and 82.48 [*SD* = 16.96], respectively). Indeed, the present mean utilities approximate to those reported in adults in the general population aged 75 years and over (0.73 [*SD* = 0.27] and 73.66 [*SD* = 18.63], respectively; Kind et al., 1999) highlighting the burden of adult ADHD in this sample of U.K. residents. These results are consistent with the findings of a study of 148 Norwegian adults aged 50 years and older diagnosed with ADHD in late adulthood, who reported significantly worse HRQoL in every EQ-5D dimension compared with an age- and gender-matched Danish population sample (Lensing et al., 2015).

The present EQ-5D scores are, however, numerically higher (indicating better HRQoL) than those obtained using the U.K. preference values (Dolan et al., 1995) by Karlsdotter et al. (2016) in a cross-sectional, observational study of 349 psychiatric outpatients with *DSM-5* adult ADHD (median age 33 years, 51.6% male) in several European countries (mean EQ-5D utility and VAS scores of 0.609 [*SD* = 0.33] and 62.0 [*SD* = 22.86], respectively), and by van Hout et al. (2012) for a cohort of 69 U.K. patients with adult ADHD (mean age 34.3 years, 46% male) who were part of the population used to develop the EuroQol mapping function (mean EQ-5D utility and VAS scores of 0.59 [*SD* = 0.33] and 63 [*SD* = 21], respectively; van Hout et al., 2012). One explanation for this difference may be that the present study excluded individuals with major mental health disorders other than ADHD, whereas Karlsdotter et al. reported a high rate of psychiatric comorbidities (88.5%), and van Hout et al. implemented a screening question designed to filter out relatively healthy patients. A number of other studies have observed that the negative impact of ADHD on HRQoL may be further exacerbated by, or may increase the risk of, psychiatric comorbidities such as anxiety and depression (Coghill, Danckaerts, Sonuga-Barke, Sergeant, & ADHD European Guidelines Group, 2009; Danckaerts et al., 2010; Kessler et al., 2006).

As already mentioned, the use of a single, generic HRQoL instrument enables different conditions to be compared. The present EQ-5D-derived mean utility and VAS values are similar to the overall mean scores (0.73 and 68.54, respectively) self-reported by patients with selected chronic conditions from National Health Service England’s primary care Quality Outcomes Framework (QOF) incentive program: asthma, chronic obstructive pulmonary disease, diabetes, epilepsy, heart failure, and stroke (Peters et al., 2014). They are also within the ranges reported in a systematic review of HRQoL in adults with psoriasis (utility, .52 to .9; VAS score, 50.7 to 75.1; Moller, Erntoft, Vinding, & Jemec, 2015) but are higher than those reported for spinal complaints (mean utility .39 [median .52]; McDonough et al., 2005).

Greater impairments in HRQoL (i.e., lower EQ-5D utilities) were associated with greater ADHD symptom severity; greater health-related impairments in work and regular daily activities; not working; having chronic medical comorbidities; being diagnosed by a psychiatry, psychology, or other service (vs. a primary care clinic or pediatric service); older age at first or second diagnosis; lack of formal qualifications; and older current age. After adjusting for gender and the presence of chronic medical comorbidities, increasing age was associated with decreasing utility and VAS score, which worsened by 0.05 and 1.77 points, respectively, for each additional decade. Increasing age has also been found to be associated with worsening EQ-5D scores in the U.K. population norms (Kind et al., 1999). In addition, telephone interviews with a small sample (*n* = 24) of adults of mean age 66 years diagnosed with ADHD later in life indicated that impairments associated with ADHD in the professional, economic, social, and emotional domains accumulated with time (Brod, Schmitt, et al., 2012). Together, these data indicate that HRQoL continues to decline with increasing age in individuals with ADHD.

This study presents self-reported EQ-5D utilities for adult ADHD. As recently as 2014, no such data had been published, according to literature reviews (Matza et al., 2014; Van Brunt, Matza, Classi, & Johnston, 2011), which led Matza et al. (2014) to develop three health state descriptions for use in adult ADHD cost–utility models. The health states were based on a literature review, clinician interviews, and clinical trial data; did not include comorbid mental health disorders; and included a statement about whether a (nonspecific) ADHD treatment was being received. Mean utility for these health states, as rated by a sample of 158 U.K. adults from the general population using time trade-off methodology, was worse for health states describing ADHD treatment nonresponders (0.68 [*SD* = 0.28]) or untreated patients (0.67 [*SD* = 0.28]) than for the state describing treatment responders (0.82 [*SD* = 0.17]) (Matza et al., 2014). Although the 2008 NICE *Guide to the methods of technology appraisal* stated that the methodology employed by Matza et al. is an acceptable way of generating EQ-5D utility values when self-reported utility is unavailable (NICE, 2008b), this provision was removed in the 2013 edition (NICE, 2013), making the present study even more relevant and timely.

The observed WPAI:GH mean impairments due to health problems of 45.65% in overall work productivity and 45.79% in regular daily activities in adults with ADHD are substantial and higher than those reported for type 2 diabetes mellitus (11.93% and 27.01%, respectively; Bays, Fox, & Grandy, 2014). The present values, however, are lower than the mean values observed by Able, Haynes, and Hong (2014) in a web-based cross-sectional survey of adults from Europe and the United States reporting an ADHD diagnosis (60.8% [*SD* = 30.4] and 55.5% [*SD* = 27.0], respectively, in the U.K. subgroup [*n* = 101]). A likely explanation could again be the high rate of psychiatric comorbidities in the population recruited by Able et al., with 76.2% of the U.K. subgroup reporting at least one of depression, anxiety/generalized anxiety disorder, sleep difficulties/insomnia, and other anxiety disorders (Able et al., 2014). In contrast, individuals reporting major psychiatric comorbidities were excluded from the present study.

The results of this study indicated that greater symptom severity, as measured by ADHD-RS-IV total score, was significantly associated with greater WPAI:GH health-related impairments in regular daily activities (*r* = .256, *p* < .001). This result complements findings from a smaller U.S. study of individuals who met *DSM-IV* criteria for ADHD in adulthood and were receiving medication (*n* = 105), in which significant associations between ADHD-RS-IV symptom severity and the work, recreation, and interpersonal domains of the Range of Impaired Functioning Tool (LIFE-RIFT) instrument were reported (Safren, Sprich, Cooper-Vince, Knouse, & Lerner, 2010). In addition, each of the WPAI:GH work-related outcomes in the present study was significantly independently associated with EQ-5D utility quartile after adjusting for age, gender, and the presence of chronic medical comorbidities. This indicates the importance of HRQoL to work and productivity and also indicates that both are impaired in adults with ADHD.

Chronic medical comorbidities were reported by around 40% of participants in this study (Table 2). The most common was asthma, which was present in more than 20% of the study population, higher than the estimated age-standardized point prevalence of clinician-diagnosed asthma in the general U.K. population aged 16 years and above (~4%-12%), and the lifetime prevalence of ~11% to 17% (Nwaru et al., 2015). This association of asthma with ADHD is well established (Instanes, Klungsoyr, Halmoy, Fasmer, & Haavik, 2016) and is consistent with the findings of a large cross-sectional Norwegian study (*N* = 1313), in which self-reported asthma prevalence was significantly higher in adults with ADHD than in those without (24.4% vs. 11.3%, odds ratio 2.53, 95% CI [1.88, 3.41]; Fasmer, Halmoy, Eagan, Oedegaard, & Haavik, 2011). Other chronic medical comorbidities reported by more than one individual in the present study were rheumatoid arthritis (3.0% of participants), diabetes (1.7%), and angina (1.3%), while 49 (21.0%) reported a chronic health problem other than those listed in the questionnaire (Table 2). However, care should be taken in extrapolating these results to the general U.K. adult ADHD population, as individuals with psychiatric comorbidities were excluded from the present study. For example, many medical comorbidities, including asthma and diabetes, are significantly more prevalent in adults with ADHD and comorbid depression than in those without depression, according to an analysis of U.S. employer-sponsored health plan data (*N* = 29,965; Hodgkins, Montejano, Sasane, & Huse, 2011).

Key strengths of this study include the use of the generic EQ-5D HRQoL instrument which enables comparison of health states across conditions and treatments, the fact that EQ5D values for adult ADHD were self-reported rather than modeled, and the inclusion of instruments that permit associations between HRQoL, health-related impairments in work and regular daily activities, and symptom severity to be investigated. In addition, the web-based survey format of the study enabled the recruitment of a diverse population of U.K. residents with adult ADHD.

A number of caveats should, however, be considered when interpreting the present data. First, the sample included 233 participants, fewer than the original target of 300. This level of recruitment allows for the identification of statistically significant differences, at the 5% level, between two groups of participants of equal size equivalent to a moderate effect size of ≈ 0.35 *SDs* (Cohen, 1988). In the whole sample, this provides a CI of maximum width ±5.5% around a percentage. The lower level of recruitment was thus considered unlikely to impact the robustness of the results. A second caveat of the study is a discrepancy over the age at which symptoms began for some participants. For a diagnosis of adult ADHD, *DSM-5* requires that several inattentive and/or hyperactive-impulsive symptoms be present before the age of 12 years (American Psychiatric Association, 2013), and at screening all 233 participants reported that symptoms had been present since primary school (typically completed before 12 years of age in the UK; Government Digital Service, 2017a, 2017b). In the subsequent survey, however, 29 participants reported that symptoms began when aged 13 to 17 years [*n* = 21, 9.0%] or at 18 years or over (*n* = 8, 3.4%). Given the additional context about challenging situations (which were aligned with the *DSM-IV/5* items) during screening, it might be likely that these participants did, in fact, experience ADHD symptoms before the age of 12 years. This is consistent with research showing that differences in context (Mathiowetz, 2000) and in wording or response format (Lee, Mathiowetz, & Tourangeau, 2007) can contribute to discrepancies in responses to disability questionnaires. A third caveat of the study is that the sample is unlikely to be fully representative of all U.K. residents with adult ADHD. For example, although similar numbers of men and women accessed the initial screening form, more men closed their web browser prematurely and more women completed the study. In addition, the mean ADHD-RS-IV total score of 43.46 indicates that study participants were toward the severe end of the ADHD symptom spectrum.

Finally, and most importantly, adult ADHD often coexists with psychiatric comorbidities (Biederman, Monuteaux, et al., 2006; Fayyad et al., 2007; Kessler et al., 2006) and the exclusion of mental health illnesses other than ADHD limits direct extrapolation of the present data to the wider adult ADHD population. On the contrary, this approach clarifies the impact of ADHD on HRQoL independently from comorbid mental health disorders, in keeping with European Medicines Agency guidelines stating that patients should not be included in clinical trials for ADHD medications if they have severe comorbid anxiety or depression, a primary *DSM-IV* Axis II disorder, or a recently diagnosed comorbid Axis I disorder (European Medicines Agency, 2010).

In summary, the findings of this study isolate and highlight the substantial burden of ADHD on the HRQoL and productivity of adults in the United Kingdom. For the first time, EQ-5D utility is reported for adult ADHD without comorbid mental health conditions and is demonstrated to be significantly associated with ADHD symptom severity and to be significantly independently associated with WPAI:GH measures of impairment to work and daily activities due to health problems after controlling for age, gender, and chronic medical comorbidities. Given the burden of adult ADHD on patients, their families, and society, physicians, and other stakeholders should seek to implement treatment strategies that improve HRQoL and minimize the impact of the disorder on work and daily activities, in addition to reducing ADHD symptoms.
